# Association of socioeconomic deprivation with outcomes in critically ill adult patients: an observational prospective multicenter cohort study

**DOI:** 10.1186/s13613-024-01279-1

**Published:** 2024-04-09

**Authors:** Morgan Benaïs, Matthieu Duprey, Laura Federici, Michel Arnaout, Pierre Mora, Marc Amouretti, Irma Bourgeon-Ghittori, Stéphane Gaudry, Pierre Garçon, Danielle Reuter, Guillaume Geri, Bruno Megarbane, Jordane Lebut, Armand Mekontso-Dessap, Jean-Damien Ricard, Daniel da Silva, Etienne de Montmollin

**Affiliations:** 1 Service de Médecine Intensive - Réanimation, Hôpital Delafontaine, Saint-Denis, France; 2 Service de Réanimation, Grand Hôpital de l’Est Francilien-Site de Marne-la-Vallée, Jossigny, France; 3 Service de Réanimation Polyvalente, Centre Hospitalier Sud Francilien, Corbeil-Essonnes, France; 4 Service de Médecine Intensive - Réanimation, AP-HP, Hôpital Ambroise Paré, Boulogne, France; 5 Service de Médecine Intensive - Réanimation, AP-HP, Hôpital Lariboisière, Paris, France; 6 Service de Réanimation Polyvalente, Groupe Hospitalier Nord-Essonne, Longjumeau, France; 7 Service de Médecine Intensive - Réanimation, AP-HP, Hôpital Henri Mondor, Créteil, France; 8 DMU ESPRIT, Service de Médecine Intensive - Réanimation, AP-HP, Hôpital Louis Mourier, Colombes, France; 9 IAME, Université Paris Cité and Université Sorbonne Paris Nord, Inserm, 75018 Paris, France; 10 Service de Médecine Intensive - Réanimation Infectieuse, AP-HP, Hôpital Bichat-Claude Bernard, 46 rue Henri Huchard, 75018 Paris, France

**Keywords:** Social deprivation, Socioeconomic factor, Socioeconomic status, Critical illness, Intensive care units

## Abstract

**Background:**

The influence of socioeconomic deprivation on health inequalities is established, but its effect on critically ill patients remains unclear, due to inconsistent definitions in previous studies.

**Methods:**

Prospective multicenter cohort study conducted from March to June 2018 in eight ICUs in the Greater Paris area. All admitted patients aged ≥ 18 years were enrolled. Socioeconomic phenotypes were identified using hierarchical clustering, based on education, health insurance, income, and housing. Association of phenotypes with 180-day mortality was assessed using Cox proportional hazards models.

**Results:**

A total of 1,748 patients were included. Median age was 62.9 [47.4–74.5] years, 654 (37.4%) patients were female, and median SOFA score was 3 [1–6]. Study population was clustered in five phenotypes with increasing socioeconomic deprivation. Patients from phenotype A (n = 958/1,748, 54.8%) were without socioeconomic deprivation, patients from phenotype B (n = 273/1,748, 15.6%) had only lower education levels, phenotype C patients (n = 117/1,748, 6.7%) had a cumulative burden of 1[1–2] deprivations and all had housing deprivation, phenotype D patients had 2 [1–2] deprivations, all of them with income deprivation, and phenotype E patients (n = 93/1,748, 5.3%) included patients with 3 [2–4] deprivations and included all patients with health insurance deprivation. Patients from phenotypes D and E were younger, had fewer comorbidities, more alcohol and opiate use, and were more frequently admitted due to self-harm diagnoses. Patients from phenotype C (predominant housing deprivation), were more frequently admitted with diagnoses related to chronic respiratory diseases and received more non-invasive positive pressure ventilation. Following adjustment for age, sex, alcohol and opiate use, socioeconomic phenotypes were not associated with increased 180-day mortality: phenotype A (reference); phenotype B (hazard ratio [HR], 0.85; 95% confidence interval CI 0.65–1.12); phenotype C (HR, 0.56; 95% CI 0.34–0.93); phenotype D (HR, 1.09; 95% CI 0.78–1.51); phenotype E (HR, 1.20; 95% CI 0.73–1.96).

**Conclusions:**

In a universal health care system, the most deprived socioeconomic phenotypes were not associated with increased 180-day mortality. The most disadvantaged populations exhibit distinct characteristics and medical conditions that may be addressed through targeted public health interventions.

**Supplementary Information:**

The online version contains supplementary material available at 10.1186/s13613-024-01279-1.

## Introduction

Socioeconomic deprivation is a multidimensional and dynamic condition, defined by an individual’s inability to participate fully in the life of his community in the context of a limited access to society’s resources due to poverty, discrimination, or other disadvantages [1]. The best way to characterize an individual’s socioeconomic status (SES) is not consensual [2], and many surrogates are found in the literature. A single distinctive socioeconomic feature can be used, such as income [3], homelessness [4], or health insurance [5, 6]. Alternatively, scores integrating various socioeconomic aspects exist, such as the index of multiple deprivation used in the United Kingdom [7, 8] or the EPICES score used in France [9, 10]. Furthermore, socioeconomic features can also be measured at the individual level, or at the area level where the individual is living. Both metrics display similar effect size, but may highlight different pathways by which socioeconomic deprivation may be associated with adverse outcomes [11–13].

Whichever proxy is used, socioeconomic deprivation is constantly associated with health inequalities. Studies show increased prevalence and mortality from chronic diseases such as diabetes [14] or cancer [15], and acute diseases such as myocardial infarction [16, 17] or stroke [18]. Education, health insurance, income and housing are socioeconomic features that have all been individually associated with poorer outcomes in the non-critically ill population [5, 14, 16, 19]. The main reasons provided to explain such an association include overexposure to risk factors [20–23] and limited access to the healthcare system [16, 24]. In the field of intensive care medicine, the burden of socioeconomic deprivation is uncertain. While some studies have shown a positive association between socioeconomic deprivation and mortality of critically ill patients [25–28], several others have shown none [4, 10, 29–32]. These conflicting results are likely due to the diverse definitions of socioeconomic deprivation and the healthcare system and income group classification [33] of the country in which the study takes place. Defining SES and the mechanistic pathways leading to socioeconomic deprivation is complex, and the use of aggregated scores or a single socioeconomic dimension to define deprivation may be inappropriate and lead to a loss of information [2].

In this context, we sought to evaluate the epidemiology and impact of socioeconomic deprivation of patients admitted to various intensive care units of the greater Paris area, a territory of high socioeconomic contrast [34] from a high-income country [35] with universal healthcare. For the assessment of socioeconomic deprivation, we chose a novel approach based on a machine learning technique: to define SES phenotypes using an unsupervised classification algorithm, based on four core socioeconomic features (education, health insurance, income and housing).

## Methods

### Study design

We carried out a prospective, multicenter, observational cohort study in eight intensive care units (ICUs) across the greater Paris area from March 1 to June 1, 2018. The study protocol was registered with the French data protection authority (Commission Nationale de l’Informatique et des Libertés, declaration number 2122051) and received approval from an institutional review board (declaration number: 2017 A01272 51). The study protocol was registered in Clinical Trials (NCT03607019). This study follows the Strengthening the Reporting of Observational Studies in Epidemiology (STROBE) reporting guidelines.

### Patients

During the study period, all consecutive patients aged 18 years or older and admitted to one of the participating ICUs were eligible. Patients were excluded if they declined to participate to the study, had been previously included in the study from a prior ICU stay, were admitted for emergency dialysis with known end-stage renal disease, and if the socioeconomic questionnaire had not been completed during their ICU stay.

### Data collection

During their ICU stay, patients or their relatives were asked to complete a socioeconomic questionnaire assessing four core, four-level socioeconomic variables: (1) housing, (2) income, (3) health insurance, and (4) education. For non-French speaking patients, relatives or hospital translators were used whenever possible. To improve the feasibility of the clustering analysis (see the statistical analysis paragraph below), the four core socioeconomic variables were arbitrarily dichotomized as follows: (1) housing deprivation, defined as living on the street, in a shelter, hotel, or hostel, or being housed by relatives; (2) income deprivation, defined as no income or minimum welfare; (3) health insurance deprivation, defined as having none or free state medical aid; and (4) education deprivation, defined as primary education or below (International Standard Classification of Education [36] level 1 or below). The French health insurance system comprises basic statutory insurance, available to all French nationals and non-French individuals with residence permits, and free state medical aid, providing insurance for undocumented residents who entered France at least three months prior. Those with basic statutory insurance may also subscribe to supplemental, typically private and for-profit, health insurance plans.

Clinical data were prospectively collected at various time points: at admission (demographics, chronic diseases, admission features, baseline severity indexes, admission diagnosis, and admission type), at ICU discharge (need and duration of organ support, length of stay, vital status, final diagnosis), at hospital discharge (length of stay, vital status, discharge destination), and at day 180 (vital status). Main comorbidities were assessed using the modified Charlson Comorbidity Index [37], while frailty before critical illness was evaluated with the Clinical Frailty Scale [38]. Severity of illness was graded at ICU admission using the Simplified Acute Physiology Score (SAPS II) [39] and the Sequential Organ Failure Assessment (SOFA) score [40]. Final diagnoses at ICU discharge were documented using the 10th International Classification of Diseases Codes (ICD-10). For data analysis and presentation, we clustered ICD-10 codes according to the Global Burden of Diseases study classification [41], commonly employed in large population health analyses [42].

### Outcomes assessment and definition of exposure

The primary outcome was vital status at day 180, recorded through the publicly available French death registry (www.insee.fr). Secondary outcomes included vital status at ICU and hospital discharge, ICU and hospital length of stay, use of organ support therapy, final diagnosis at ICU discharge, and hospital discharge destination.

The primary exposure was SES, assessed through SES phenotypes created using an unsupervised machine learning classification algorithm (see statistical analysis section) based on the four core features recorded in the questionnaire (education, health insurance, income, and housing).

### Statistical analysis

Patients’ characteristics were described as counts and frequencies for categorical variables and medians with interquartile ranges for quantitative variables. Univariable comparisons between subgroups were performed using the Wilcoxon test for continuous variables and the Chi-square test or Fisher exact test, as appropriate, for categorical variables.

For unsupervised clustering analysis, we used the four core variables previously defined: education, health insurance, income, and housing. Missing values for these variables were handled through multiple imputation using the R package “mice”. The clustering tendency of the database (i.e., feasibility of the clustering analysis) was assessed visually with the “mclust” and “factoextra” R packages. Clustering tendency improved when treating socioeconomic variables as binary variables. Optimal clusterization technique and number of clusters were determined by the Dunn index using the "clValid" R package, suggesting a hierarchical clustering technique using five clusters. After clusterization, validity was assessed by the silhouette coefficient using the “fpc” R package to identify patients with suboptimal clustering.

To assess the association of the five phenotypes produced by clustering analysis with 180-day mortality in a causal inference framework, we used a Cox proportional hazard model adjusted on confounders identified through the use of a Directed Acyclic Graph (DAG) (see Additional file 1: Fig. S1) [43]. In a total effect estimation model, we included only variables identified as confounders (a variable associated with the exposure of interest, a cause of the outcome of interest, and that does not reside in the causal pathway between the exposure and outcome): age, sex, alcohol and opiate use. In an attempt to identify a direct causal pathway between socioeconomic phenotypes and 180-day mortality, we also performed a direct effect estimation model, also including non-colinear mediators (a variable that lies along the causal path between the exposure and the outcome): admission SOFA score and the Charlson comorbidity index. The models were stratified by inclusion center. Post-hoc sensitivity analyses were conducted: (1) we assessed the association of each separate socioeconomic deprivation with 180-day mortality, (2) we assessed the association of cumulative socioeconomic deprivation with 180-day mortality, (3) we assessed the association of socioeconomic clusters with 180-day mortality after reclassification of patients with suboptimal clustering characteristics to the nearest neighbor cluster and (4) we assessed the association of socioeconomic clusters with 180-day mortality without adjusting on alcohol and opiate use.

For all other variables, missing data completely at random with less than 10% missing values were handled by simple imputation with the median/most frequent method [44]. The DAG was created using the DAGitty v3.1 software (https://dagitty.net/dags.html). A *P* value of 0.05 or lower was considered statistically significant. All statistical analyses were conducted with SAS 9.4 (SAS Institute Inc) and R version 3.5.2 (R Foundation for Statistical Computing).

## Results

### Study population

Between March 1st and June 1st, 2018, we screened 2,006 ICU patients from eight ICUs. Among them, 22 patients younger than 18 years were not included, and 236 patients were excluded, primarily for not having completed the socioeconomic questionnaire (n = 129) (see Additional file 1: Fig. S2). Ultimately, 1,789 patients were included in the analysis; median age was 62.9 [47.4–74.5] years and 654 (37%) were female. Baseline characteristics of the included patients are presented in Table 1.

**Table 1:** Baseline characteristics of patients at presentation to the ICU

Characteristics	Patients, No/total No (%)						*P* value
	All patients, n = 1748	Socioeconomic phenotypes					
		A, n = 958	B, n = 273	C, n = 117	D, n = 307	E, n = 93	
Demographics							
Age, median [IQR], y	62.9 [47.4–74.5]	66.2 [52.6–75.6]	70.2 [61.7–80.7]	62.1 [33.4–75.3]	49 [30.5–58.5]	49 [34.9–59.9]	< 0.001
Female sex	654/1748 (37.4)	361/958 (37.7)	100/273 (36.6)	44/117 (37.6)	120/307 (39.1)	29/93 (31.2)	0.73
Preadmission status							
Clinical Frailty Scale score ≥ 5	388/1748 (22.2)	206/958 (21.5)	72/273 (26.4)	30/117 (25.6)	71/307 (23.1)	9/93 (9.7)	0.01
Charlson comorbidity index ≥ 1	976/1733 (56.3)	569/953 (59.7)	171/272 (62.9)	59/116 (50.9)	143/303 (47.2)	34/89 (38.2)	< 0.001
Chronic obstructive pulmonary disease	250/1745 (14.3)	146/955 (15.3)	43/273 (15.8)	26/117 (22.2)	28/307 (9.2)	7/93 (7.6)	0.01
Congestive heart failure	232/1743 (13.3)	140/952 (14.7)	44/273 (16.1)	20/117 (17.1)	23/307 (7.5)	5/93 (5.4)	0.01
Diabetes mellitus	438/1742 (25.1)	231/954 (24.2)	94/273 (34.4)	29/117 (24.8)	72/306 (23.5)	12/90 (13.3)	< 0.001
Chronic kidney disease	207/1744 (11.9)	116/958 (12.1)	41/273 (15)	15/117 (12.8)	24/307 (7.8)	11/91 (12)	0.11
Cirrhosis	62/1739 (3.6)	29/958 (3)	12/272 (4.4)	1/115 (0.9)	14/305 (4.6)	6/90 (6.7)	0.12
Cancer	198/1741 (11.4)	133/957 (13.9)	31/272 (11.4)	8/116 (6.9)	17/304 (5.6)	9/92 (9.8)	0.01
Alcohol use	340/1748 (19.5)	183/958 (19.1)	45/273 (16.5)	16/117 (13.7)	66/307 (21.5)	30/93 (32.3)	< 0.001
Opioid use	39/1748 (2.2)	9/958 (0.9)	2/273 (0.7)	3/117 (2.6)	22/307 (7.2)	3/93 (3.2)	< 0.001
Current smoker	492/1748 (28.1)	254/948 (26.5)	53/273 (19.4)	34/117 (29.1)	116/307 (37.8)	35/93 (37.6)	< 0.001
BMI, median [IQR], kg/m2	24.9 [21.6–29.3]	25.3 [22.2–29.4]	25.8 [22.5–30.4]	25.1 [21.2–28.9]	23.9 [20.1–28.1]	23.5 [20.7–27.8]	< 0.001
ICU admission status							
Direct ICU admission from ED or home	1080/1748 (61.8)	575/958 (60)	167/273 (61.2)	83/117 (70.9)	192/307 (62.5)	63/93 (67.7)	0.14
Time from hospital to ICU admission, median [IQR], d	0 [0–1]	0 [0–1]	0 [0–1]	0 [0–0]	0 [0–1]	0 [0–0]	0.03
Reason for ICU admission							
Respiratory failure	490/1748 (28)	259/958 (27)	88/273 (32.2)	47/117 (40.2)	73/307 (23.8)	23/93 (24.7)	< 0.001
Shock	176/1748 (10.1)	110/958 (11.5)	25/273 (9.2)	4/117 (3.4)	26/307 (8.5)	11/93 (11.8)	
Coma of cerebrovascular cause	26/1748 (1.5)	16/958 (1.7)	8/273 (2.9)	0/117 (0)	1/307 (0.3)	1/93 (1.1)	
Coma of toxic cause	120/1748 (6.9)	57/958 (5.9)	10/273 (3.7)	8/117 (6.8)	32/307 (10.4)	13/93 (14)	
Coma of other cause	93/1748 (5.3)	42/958 (4.4)	11/273 (4)	7/117 (6)	27/307 (8.8)	6/93 (6.5)	
Cardiac arrest	105/1748 (6)	68/958 (7.1)	15/273 (5.5)	2/117 (1.7)	14/307 (4.6)	6/93 (6.5)	
Other	738/1748 (42.2)	406/958 (42.4)	116/273 (42.5)	49/117 (41.9)	134/307 (43.6)	33/93 (35.5)	
SAPS II without age, median [IQR], pts	35 [22–50]	24 [15–38]	24 [15–40]	22 [12–32]	24 [13–40]	25 [15–38]	0.20
SOFA score, median [IQR], pts	3 [1–6]	3 [2–6]	3 [2–6]	3 [1–5]	3 [1–6]	4 [1–7]	0.07

*BMI* body mass index; *d* days; *ED* emergency department; *ICU* intensive care unit; *IQR* interquartile range; *SAPS* simplified acute physiology score; *SOFA* sequential organ failure assessment; *y* years; *pts* points

Detailed socioeconomic characteristics can be found in Additional file 1: Table S1. Deprived housing was present in 317/1726 (18.4%) patients, deprived health insurance in 93/1724 (5.4%) patients, deprived income in 352/1700 (20.7%) patients, and deprived education in 428/1595 (26.8%) patients. Overall, 1326/1748 (75.9%) patients were French nationals, and 75/1748 (4.3%) patients were undocumented migrants. We observed considerable variability in the frequency of deprivation features among enrollment centers, which correlated with the poverty rate of the area where the hospital was located (see Additional file 1: Table S2 and Fig. S3).

Patients were admitted to the ICU directly from the emergency department or home in 1080/1748 (61.8%) cases, primarily for respiratory failure (n = 490/1,748, 28%), coma (n = 239/1748, 13.7%), and shock (n = 176/1748, 10.1%) (Table 1). Admission SAPS II without age and SOFA scores were 35 [22–50] and 3 [1–6], respectively. Details on ICU management and outcomes are presented in Table 2. ICU, hospital, and day-180 mortality were 222/1,748 (12.7%), 274/1,748 (15.7%), and 404/1,748 (23.1%), respectively.

**Table 2:** ICU Management and Clinical Outcomes

Characteristics	Patients No/total No (%)						*P* value
	All patients, n = 1748	Socioeconomic phenotypes					
		A, n = 958	B, n = 273	C, n = 117	D, n = 307	E, n = 93	
Supportive care							
Use of NIPPV	297/1748 (17)	156/958 (16.3)	50/273 (18.3)	32/117 (27.4)	48/307 (15.6)	11/93 (11.8)	0.02
Use of HFNO	141/1748 (8.1)	75/958 (7.8)	21/273 (7.7)	15/117 (12.8)	24/307 (7.8)	6/93 (6.5)	0.40
Use of invasive ventilation	530/1748 (30.3)	295/957 (30.8)	85/273 (31.3)	20/117 (17.1)	97/307 (31.6)	33/93 (35.5)	0.02
Duration of invasive ventilation, median [IQR], d	3 [1–7]	3 [1–7]	3 [1–6]	2 [1–9.5]	2 [1–6]	2 [1 2]	0.02
Use of vasopressors	424/1745 (24.3)	264/957 (27.6)	69/272 (25.4)	19/117 (16.2)	52/307 (16.9)	20/93 (21.5)	< 0.001
Need for RRT	149/1747 (8.5)	91/957 (9.5)	22/273 (8.1)	9/117 (7.7)	16/307 (5.2)	11/93 (11.8)	0.14
Need for ECLS	32/1746 (1.8)	24/958 (2.5)	1/272 (0.4)	0 (0)	4/306 (1.3)	3/93 (3.2)	0.05
Diagnosis at ICU discharge							
Communicable maternal neonatal and nutritional diseases	279/1656 (16.8)	151/904 (16.7)	45/255 (17.6)	14/111 (12.6)	47/294 (16)	22/91 (24.2)	0.11
Non-communicable diseases	1007/1656 (60.8)	197/904 (21.8)	47/255 (18.4)	27/111 (24.3)	72/294 (24.5)	27/91 (29.7)	
Injuries	370/1656 (22.3)	557/904 (61.5)	163/255 (63.9)	70/111 (63.1)	175/294 (59.5)	42/91 (46.2)	
Outcomes							
Decision of WLST	240/1746 (13.7)	155/957 (16.2)	40/273 (14.7)	14/117 (12)	26/307 (8.5)	5/93 (5.4)	< 0.001
Time from ICU admission to WLST, median [IQR], d	3 [1–6]	3 [1–7]	3 [1–6]	2 [1–9.5]	2 [1–6]	2 [1 2]	0.37
ICU length of stay, median [IQR], d	4 [3–7]	4 [3–8]	4 [3–7]	5 [3–7]	4 [3–7]	3 [2–5]	< 0.001
Hospital length of stay, median [IQR], d	9 [4–18]	9.5 [4–18]	11 [5–19]	9 [4–21]	8 [4–19]	6 [3–12]	< 0.001
Destination at hospital discharge							
Home	923/1463 (63.1)	479/787 (60.9)	139/220 (63.2)	65/109 (59.6)	178/268 (66.4)	62/79 (78.5)	0.003
Other acute care hospital	360/1463 (24.6)	192/787 (24.4)	51/220 (23.2)	29/109 (26.6)	73/268 (27.2)	15/79 (19)	
Sub-acute care and rehabilitation facility	159/1463 (10.9)	109/787 (13.9)	24/220 (10.9)	9/109 (8.3)	15/268 (5.6)	2/79 (2.5)	
Long-term care facility	21/1463 (1.4)	7/787 (0.9)	6/220 (2.7)	6/109 (5.5)	2/268 (0.7)	0/79 (0)	
Mortality							
In ICU	222/1748 (12.7)	142/958 (14.8)	37/273 (13.6)	6/117 (5.1)	27/307 (8.8)	10/93 (10.8)	0.002
In hospital	274/1748 (15.7)	169/958 (17.6)	49/273 (17.9)	8/117 (6.8)	34/307 (11.1)	14/93 (15.1)	0.001
At day 180	404/1748 (23.1)	250/958 (26.1)	72/273 (26.4)	16/117 (13.7)	48/307 (15.6)	18/93 (19.4)	0.003

*d* days; *ECLS* extra corporeal life support; *HFNO* high flow nasal oxygen; *ICU* intensive care unit; *IQR* interquartile range; *NIPPV* non-invasive positive pressure ventilation; *RRT* renal replacement therapy; *WLST* withdrawal or withholding of life sustaining therapies

### Population clustering

**Fig. 1:**
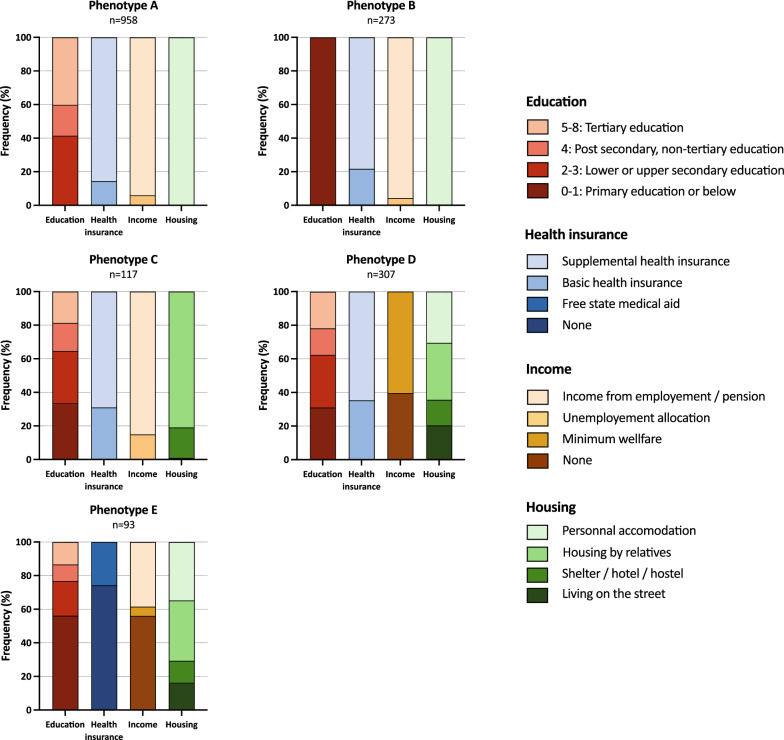
Characterization of Socioeconomic Phenotypes After Hierarchical Clustering

Hierarchical clustering analysis grouped the study population into five phenotypes, labeled A to E, and ordered by increasing cumulative deprivation (Fig. 1 and Additional file 1: Table S1 and Fig. S4). Validity assessment identified only 26/1748 (1.5%) patients with suboptimal clustering. Patients from phenotypes A (n = 958/1748, 54.8%) had no socioeconomic deprivation, phenotype B (n = 273/1748, 15.6%) patients only had lower education levels, patients from phenotype C (n = 117/1748, 6.7%) had an intermediate cumulative burden of 1 [1, 2] deprivations and all had housing deprivation, patients from phenotype D (n = 307/1748, 17.6%) had an intermediate cumulative burden of 2 [1–2] deprivations and all had income deprivation, and phenotype E (n = 93/1748, 5.3%) included patients with a high cumulative burden of 3 [2–4] deprivations and included all patients with health insurance deprivation. Although the majority of patients from phenotype E were undocumented migrants, 18 out of 93 (19.4%) were French nationals. Phenotype case mix varied across enrollment centers and is presented in Additional file 1: Fig. S5.

Table 1 presents the study population characteristics according to SES phenotypes. Compared to phenotypes A, B and C, patients from phenotypes D and E were younger (A, 66.2 [52.6–75.6] vs B, 70.2 [61.7–80.7] vs C, 62.1 [33.4–75.3] vs D, 49 [30.5–58.5] vs E, 49 [34.9–59.9] years; *P* < 0.001), and had fewer Charlson comorbidity indexes ≥ 1 (A, 569/953 (59.7%) vs B, 171/272 (62.9%) vs C, 59/116 (50.9%) vs D, 143/303 (47.2%) vs E, 34/89 (38.2%); *P* < 0.001). Alcohol use was most frequent in phenotype E (A, 183/958 (19.1%) vs B, 45/273 (16.5%) vs C, 16/117 (13.7%) vs D, 66/307 (21.5%) vs E, 30/93 (32.3%); *P* < 0.001) while opiate use was more frequent in phenotype D (A, 9/958 (0.9%) vs B, 2/273 (0.7%) vs C, 3/117 (2.6%) vs D, 22/307 (7.2%) vs E, 3/93 (3.2%); *P* < 0.001). Phenotypes D and E were more frequently admitted to the ICU for coma of toxic cause (A, 57/958 (5.9%) vs B, 10/273 (3.7%) vs C, 8/117 (6.8%) vs D, 32/307 (10.4%) vs E, 13/93 (15%)), while phenotype C patients were admitted predominantly for respiratory failure (A, 259/958 (27%) vs B, 88/273 (32.2%) vs C, 47/117 (40.2%) vs D, 73/307 (23.8%) vs E, 23/93 (24.7%)). SOFA and SASP II scores without age at ICU admission were similar across phenotypes.

### Association of socioeconomic phenotypes with the primary outcome

In the multivariable total effect estimation model, after adjusting for age, sex, and alcohol and opiate use, and compared to phenotype A (no socioeconomic deprivation), no socioeconomic phenotype was associated with increased day-180 mortality. Phenotype C, on the contrary, was associated with improved day-180 mortality (compared to A, hazard ratio (HR), 0.56; 95% confidence interval (CI), 0.34–0.93) (Table 3). In the direct effect estimation model including additional adjustment for Charlson comorbidity index and admission SOFA score, phenotype C was no longer associated with improved day-180 mortality (HR 0.69, CI 0.41 − 1.16). Post-hoc sensitivity analyses showed similar results in both total effect and direct effect models (see Additional file 1: Table S3, S4, S5 and S6).

**Table 3:** Multivariable analyses of factors associated with 180-day mortality^a^

	Total effect estimation model		Direct effect estimation model	
Variable	HR [95% CI]	*P* value	HR [95% CI]	*P* value
Socioeconomic phenotype		0.13		0.56
Phenotype A	Reference		Reference	
Phenotype B	0.85 [0.65–1.12]		0.94 [0.71–1.24]	
Phenotype C	0.56 [0.34–0.93]		0.69 [0.41–1.16]	
Phenotype D	1.09 [0.78–1.51]		0.98 [0.70–1.35]	
Phenotype E	1.20 [0.73–1.96]		1.24 [0.75–2.03]	
Age per 1 year increment	1.04 [1.04–1.05]	< 0.001	1.03 [1.03–1.04]	< 0.001
Female sex	0.63 [0.51–0.78]	<0 .001	0.83 [0.66–1.04]	0.11
Alcohol or opiate use	0.91 [0.70–1.18]	0.47	0.89 [0.68–1.17]	0.41
Charlson comorbidity index ≥ 1	–	–	1.34 [1.05–1.71]	0.02
Admission SOFA score, per point	–	–	1.22 [1.20–1.25]	< .001

*CI* confidence interval; *HR* hazard ratio; *SOFA* sequential organ failure assessment

^a^A cox proportional hazard model stratified on inclusion center was applied

### Association of socioeconomic phenotypes with the secondary outcomes

Univariate analysis of ICU management and clinical outcomes is presented in Table 2. Patients from phenotype C received more non-invasive positive pressure ventilation (NIPPV) (A, 156/958 (16.3%) vs B, 50/273 (18.3%) vs C, 32/117 (27.4%) vs D, 48/307 (15.6%) vs E, 11/93 (11.8%), *P* = 0.02) and less invasive ventilation and vasopressors. Patients from phenotype D and E received fewer decisions of withdrawal or withholding of life sustaining therapies (A, 155/957 (16.2%) vs B, 40/273 (14.7%) vs C, 14/117 (12%) vs D, 26/307 (8.5%) vs E, 5/93 (5.4%), *P* < 0.001). Hospital length of stay was shorter for patients from phenotype E (A, 9.5 [4–18] vs B, 11 [5–19] vs C, 9 [4–21] vs D, 8 [4–19] vs E, 6 [3–12] days, *P* < 0.001), with more frequent direct discharge to the home (A, 479/787 (60.9%) vs B, 139/220 (63.2%) vs C, 65/109 (59.6%) vs D, 178/268 (66.4%) vs E 62/79 (78.5%), *P* = 0.003).

**Fig. 2:**
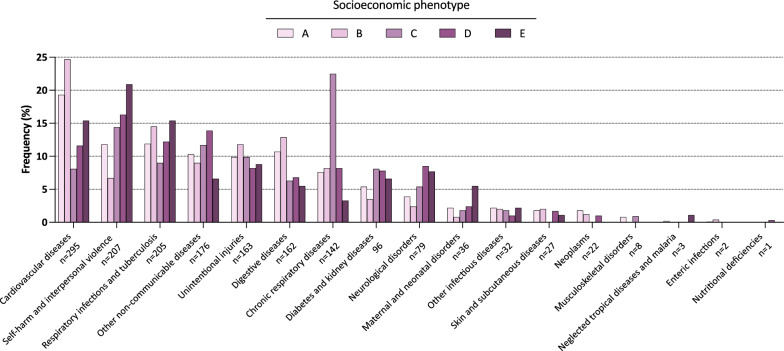
Diagnosis at ICU Discharge According to Socioeconomic Phenotypes

Complete diagnoses at ICU discharge according to SES phenotypes are presented in Fig. 2, and Additional file 1: Tables S7, S8 and S9. ICU stays from phenotypes with increasing deprivation were more frequently associated with diagnoses related to self-harm (A, 107/905 (11.8%) vs B, 17/255 (6.7%) vs C, 16/111 (14.4%) vs D, 48/294 (16.3%) vs E, 19/91 (20.9%), *P* < 0.01) and maternal disorders (A, 20/905 (2.2%) vs B, 2/255 (0.8%) vs C, 2/111 (1.8%) vs D, 7/294 (2.4%) vs E, 5/91 (5.5%), *P* = 0.13). ICU stays from phenotype C, on the other hand, were more frequently associated with diagnoses related to chronic respiratory diseases (A, 69/905 (7.6%) vs B, 21/255 (8.2%) vs C, 25/111 (22.5%) vs D, 24/294 (8.2%) vs E, 3/91 (3.3%), *P* < 0.01).

## Discussion

In this prospective multicenter cohort study of 1,748 critically ill patients conducted in the greater Paris area, we defined five SES phenotypes using an unsupervised machine learning clustering algorithm. Patients from phenotype A were without deprivation, phenotype B patients only had lower education levels, patients from phenotype C had a cumulative burden of 1 [1–2] deprivations and all had housing deprivation, patients from phenotype D (n = 307/1748, 17.6%) had an intermediate cumulative burden of 2 [1–2] deprivations and all had income deprivation, and phenotype E (n = 93/1748, 5.3%) included patients with a high cumulative burden of 3 [2–4] deprivations and included all patients with health insurance deprivation. We found that patients from phenotypes D and E were younger, had fewer comorbidities, more alcohol and opiate use, and were more frequently admitted due to self-harm diagnoses. Furthermore, patients from phenotype E had shorter ICU stays and were more often directly discharged to their homes. After adjusting for confounding factors, socioeconomic phenotypes were not associated with increased 180-day mortality.

To the best of our knowledge, this study is the first to use an unsupervised clustering method to define SES phenotypes, which offers a practical approach to the exploration of complex relationships among multiple correlated variables [45]. We believe that this exploratory approach offers a new and complementary approach to the understanding the relationships between socioeconomic deprivation features and its impact on outcomes. Indeed, evaluating socioeconomic comparability of individuals based on education or income alone is fraught with unmeasured confounders. Braveman et al*.* suggested that SES measurement should involve plausible explanatory pathways, a maximum of relevant socioeconomic information, and specify the particular socioeconomic factors measured rather than SES overall [2]. Notably, our unsupervised statistical approach, devoid of any a priori assumptions, has generated phenotypes that are both coherent and clinically significant (Fig. 1). However, whether these phenotypes are more informative than previous modeling methods (e.g., using single factors or other deprivation index definitions) in survival analysis remains to be demonstrated.

We found no association between socioeconomic deprivation and increased mortality, in accordance with prior research on the French population [10, 29]. However, these results are in contrast with a recent meta-analysis of 38 studies showing that lower socioeconomic status was associated with higher mortality at ≤ 30 days following critical care admission [11]. The inconsistent results found in the existing literature may be attributed to varying definitions of socioeconomic deprivation, different economic settings (low-, middle- or high-income countries) and different health and social policies.

Our cohort appears highly deprived, with 25.2% of patients lacking supplemental health insurance (see Additional file 1: Table S1), compared to 3.6% for the general French population [46]. Undocumented migrants were also ten times more prevalent in our cohort than in the general French population (4.3% vs 0.4%) [47]. It is interesting to note that the algorithm has classified undocumented migrants overwhelmingly to Phenotype E, and it could be argued that this group is better defined by this characteristic than by the usual understanding of socioeconomic status. However, as 20% of patients from this phenotype were French nationals, lack of health insurance appears to be the highlight of this group. Despite French law providing health insurance to all residents (including undocumented immigrants) after three months, 4% of our cohort remained uninsured. This suggests that barriers to accessing health insurance exist, potentially compounded by other social difficulties. In the United States, lack of health insurance has been linked to increased 30-day mortality and reduced utilization of common critical care procedures [6, 26]. However, in our study, separate assessment of health insurance deprivation did not show a significant association with 180-day mortality (see Additional file 1: Table S3).

Interestingly, we also found that phenotype C, which includes patients with housing deprivation but no health insurance or income deprivation was associated with improved 180-day survival in the total effect estimation model. This finding may be due to a large proportion of patients admitted for acute respiratory failure related with a final diagnosis of chronic obstructive pulmonary disease (COPD) and asthma (Fig. 2 and Additional file 1: Figure S9), treated mainly by NIPPV (Table 2). This hypothesis is supported by the fact that this association was no longer significant in the direct effect model adjusted for comorbidities. This finding is also consistent with previously published literature showing that increasing housing deprivation is associated with increased prevalence of COPD and asthma [48].

### Strengths and limitations

Our study's primary strengths include a multicenter cohort, prospective individual assessment of socioeconomic features, and the innovative use of unsupervised hierarchical clustering to identify SES phenotypes.

However, the study does have several limitations. First, France, a high-income country with universal healthcare and robust social policies, provides a unique context that may limit the generalizability of our findings to different healthcare systems or to low- or middle-income nations. Second, as socioeconomic features were arbitrarily dichotomized, different cut-offs may have led to variations in the composition of socioeconomic clusters. Furthermore, missing values on socioeconomic features cannot be considered to be “completely at random”, and we made a methodological decision to choose multiple imputation over complete case analysis. Third, while vital status is a crucial endpoint in intensive care, outcomes such as post-ICU healthcare utilization, SES changes, and quality of life may offer more meaningful insights. Furthermore, our follow-up to 180 days after ICU admission may have been insufficient to capture the effects of social deprivation on outcome. Also, follow-up using the French death registry may have introduced a detection bias, as the most deprived patients may not have had the standardized documentation that would allow consistent registration. Fourth, as socioeconomic data were collected through a questionnaire, we cannot exclude an information bias. In addition, 129 (7%) patients admitted during the study period did not complete the socioeconomic questionnaire. We did not record the reason for not completing the questionnaire, but isolated patients may have been overrepresented among these patients, introducing a selection bias. Fifth, the study's timing, conducted during spring, may affect the applicability of our findings. Socially deprived patients' vulnerability may vary seasonally, with potentially different impacts on ICU mortality in winter. Finally, the study was conducted before the COVID pandemic, a global event that may have exacerbated socioeconomic deprivation.

## Conclusions

In this prospective, multicenter cohort study involving critically ill adults from the Greater Paris area, we defined five increasingly deprived SES phenotypes using an unsupervised clustering algorithm. The socioeconomic phenotypes were not associated with increased 180-day mortality. Our findings indicated that the most disadvantaged populations exhibit distinct characteristics and medical conditions that may be addressed through targeted public health interventions. Further investigation is required to ascertain whether these phenotypes are linked to long-term healthcare utilization and patient-centered outcomes.

### Supplementary Information

**Additional file 1: Figure S1.** Figure. Directed acyclic graph and rational for variable selection. **Figure S2.** Figure. Study flowchart. **Figure S3.** Figure. Poverty rate in the greater Paris area (2017), and location of participating centers. **Figure S4.** Figure. Frequency of socioeconomic deprivation dimensions among each phenotype, using a binary classification. **Figure S5.** Figure. Distribution of socioeconomic phenotypes according to participating center. **Table S1.** Table. Detailed socioeconomic characteristics of the study population. **Table S2.** Table. Patients’ characteristics and outcomes according to inclusion center. **Table S3.** Table. Evaluation of the association of each individual socioeconomic deprivation factor with 180-day mortality (sensitivity analysis). **Table S4.** Table. Evaluation of the association of cumulative socioeconomic deprivation with 180-day mortality (sensitivity analysis). **Table S5.** Table. Multivariable analyses of factors associated with 180-day mortality in patients with suboptimal clustering characteristics reclassified to the nearest neighboring cluster (sensitivity analysis). **Table S6.** Table. Multivariable analyses of factors associated with 180-day mortality, without adjustment on alcohol and opiate use (sensitivity analysis). **Table S7.** Table. Diagnosis of communicable, maternal, neonatal and nutritional diseases at ICU discharge for the complete population and according to socioeconomic phenotypes. **Table S8.** Table. Diagnosis of Injuries at ICU discharge for the complete population and according to socioeconomic phenotypes. **Table S9.** Table. Diagnosis of non-communicable diseases at ICU discharge for the complete population and according to socioeconomic phenotypes.

## Data Availability

The datasets used and/or analyzed during the current study are available from the corresponding author on reasonable request.

## Abbreviations

DAG: Directed acyclic graph
ICD: International classification of diseases
ICU: Intensive care unit
NIPPV: Non-invasive positive pressure ventilation
SAPS II: Simplified acute physiology score
SES: Socioeconomic phenotype
SOFA: Sequential organ failure assessment
