# Supplementary material for: Association of socioeconomic deprivation with outcomes in critically ill adult patients: an observational prospective multicenter cohort study
Source: Ann Intensive Care. 2024 Apr 9;14:54. doi: 10.1186/s13613-024-01279-1 (PMC11004098; doi:10.1186/s13613-024-01279-1)
Supplement: Supplementary file 1 — Additional file 1: Figure S1. Figure. Directed acyclic graph and rational for variable selection. Figure S2. Figure. Study flowchart. Figure S3. Figure. Poverty rate in the greater Paris area (2017), and location of participating centers. Figure S4. Figure. Frequency of socioeconomic deprivation dimensions among each phenotype, using a binary classification. Figure S5. Figure. Distribution of socioeconomic phenotypes according to participating center. Table S1. Table. Detailed socioeconomic characteristics of the study population. Table S2. Table. Patients’ characteristics and outcomes according to inclusion center. Table S3. Table. Evaluation of the association of each individual socioeconomic deprivation factor with 180-day mortality (sensitivity analysis). Table S4. Table. Evaluation of the association of cumulative socioeconomic deprivation with 180-day mortality (sensitivity analysis). Table S5. Table. Multivariable analyses of factors associated with 180-day mortality in patients with suboptimal clustering characteristics reclassified to the nearest neighboring cluster (sensitivity analysis). Table S6. Table. Multivariable analyses of factors associated with 180-day mortality, without adjustment on alcohol and opiate use (sensitivity analysis). Table S7. Table. Diagnosis of communicable, maternal, neonatal and nutritional diseases at ICU discharge for the complete population and according to socioeconomic phenotypes. Table S8. Table. Diagnosis of Injuries at ICU discharge for the complete population and according to socioeconomic phenotypes. Table S9. Table. Diagnosis of non-communicable diseases at ICU discharge for the complete population and according to socioeconomic phenotypes. [file 13613_2024_1279_MOESM1_ESM.docx]

**Additional file 1**

**Association of Socioeconomic Deprivation with Outcomes in Critically Ill Adult Patients: An observational prospective multicenter cohort study**

[Figure S1. Directed acyclic graph and rational for variable selection 2](#_Toc161412448)

[Figure S2. Study flowchart 3](#_Toc161412449)

[Figure S3. Poverty rate in the greater Paris area (2017), and location of participating centers 4](#_Toc161412450)

[Figure S4. Frequency of socioeconomic deprivation dimensions among each phenotype, using a binary classification ^a^ 5](#_Toc161412451)

[Figure S5. Distribution of socioeconomic phenotypes according to participating center 6](#_Toc161412452)

[Table S1. Detailed socioeconomic characteristics of the study population 7](#_Toc161412453)

[Table S2. Patients’ characteristics and outcomes, by inclusion center 8](#_Toc161412454)

[Table S3. Evaluation of the association of each individual socioeconomic deprivation factor with 180-day mortality ^a^ 10](#_Toc161412455)

[Table S4. Evaluation of the association of cumulative socioeconomic deprivation with 180-day mortality 11](#_Toc161412456)

[Table S5. Multivariable analyses of factors associated with 180-day mortality in patients with suboptimal clustering characteristics reclassified to the nearest neighboring cluster 12](#_Toc161412457)

[Table S6. Multivariable analyses of factors associated with 180-day mortality, without adjustment on alcohol and opiate use 13](#_Toc161412458)

[Table S7. Diagnosis of communicable, maternal, neonatal and nutritional diseases at ICU discharge for the complete population and according to socioeconomic phenotypes ^a^ 14](#_Toc161412459)

[Table S8. Diagnosis of Injuries at ICU discharge for the complete population and according to socioeconomic phenotypes ^a^ 15](#_Toc161412460)

[Table S9. Diagnosis of non-communicable diseases at ICU discharge for the complete population and according to socioeconomic phenotypes ^a^ 16](#_Toc161412461)

Figure S1. Directed acyclic graph and rational for variable selection


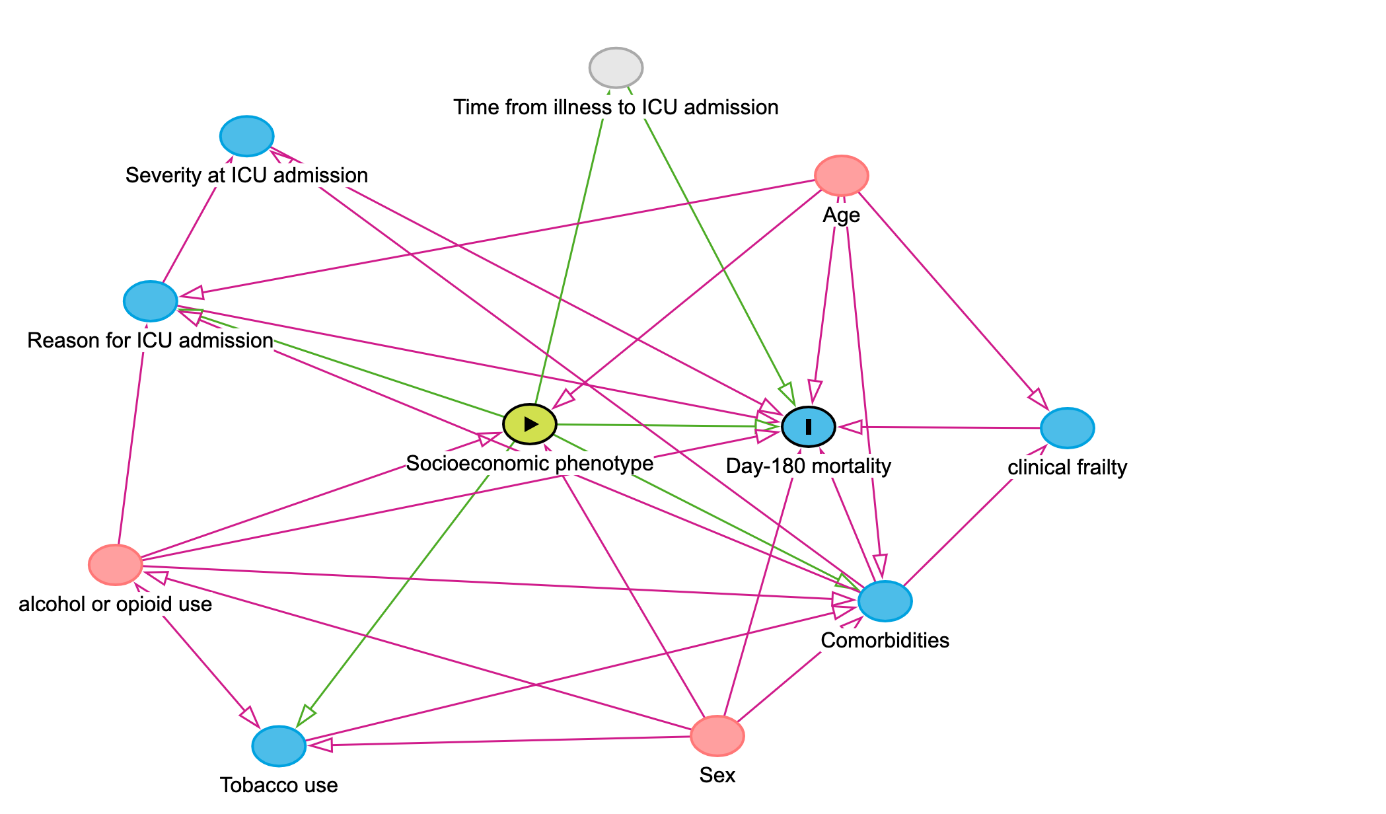


1. A confounder a variable that is associated with the exposure of interest, is a cause of the outcome of interest, and does not reside in the causal pathway between the exposure and outcome. The multivariable model should be controlled for those variables. Identified confounders in the DAG are age, sex, alcohol or opiate use.
2. A mediator is a variable that lies along the causal path between the exposure and the outcome. They are causes and mechanisms of effect of the variable of interest (socioeconomic phenotype) on the outcome (day-180 mortality). In a total effect estimation model, they should not be controlled for. However, when investigating a direct effect between the exposure of interest and the outcome of interest, they may be included in the multivariable model. According to the above DAG, the following variables were considered as mediators via an indirect causal pathway: reason for ICU admission, severity at ICU admission, time from illness to ICU admission, tobacco use, comorbidities, clinical frailty.
3. A collider is a variable where both the exposure and outcome are causes of the variable and should not be controlled for. We did not find any collider in our variable set.

The DAG was created using the DAGitty v3.1 software (<https://dagitty.net/dags.html>). Time from illness to ICU admission is a latent, unmeasured variable.

Figure S2. Study flowchart

Figure S3. Poverty rate in the greater Paris area (2017), and location of participating centers

Adapted from <https://www.insee.fr/fr/statistiques/3291402>. The poverty threshold is defined as 60% of the median income of the French population. In 2017, it was 1041€ per month.

Figure S4. Frequency of socioeconomic deprivation dimensions among each phenotype, using a binary classification ^a^

^a^ Definitions of binary socioeconomic deprivation is as follows: Housing deprivation, living on the street or shelter/hotel/hostel or housing by relatives; Income deprivation, no income or minimum welfare; Health insurance deprivation, no health insurance or free state medical aid; Education deprivation: primary education or below.

Figure S5. Distribution of socioeconomic phenotypes according to participating center

Table S1. Detailed socioeconomic characteristics of the study population

| **Characteristics** | **Patients, No/total No (%)** | | | | | |
| --- | --- | --- | --- | --- | --- | --- |
|  | **All patients**  n=1748 | **Socioeconomic phenotypes** | | | | |
|  |  | **A**  n=958 | **B**  n=273 | **C**  n=117 | **D**  n=307 | **E**  n=93 |
| Cumulative deprivation, median [IQR] | 0 [0–1] | 0 [0–0] | 1 [1–1] | 1 [1–2] | 2 [1–2] | 3 [2–4] |
| French nationality | 1326/1748 (75.9) | 830/958 (86.6) | 184/273 (67.4) | 78/117 (66.7) | 216/307 (70.4) | 18/93 (19.4) |
| Undocumented migrants | 75/1748 (4.3) | 0 (0) | 0 (0) | 0 (0) | 0 (0) | 75/93 (80.6) |
| **Education** | | | | | |  |
| Primary education or below | 428/1595 (26.8) | 0 (0) | 253/253 (100) | 41/112 (36.6) | 88/284 (31) | 46/82 (56.1) |
| Lower or upper secondary education | 496/1595 (31.1) | 358/864 (41.4) | 0 (0) | 32/112 (28.6) | 89/284 (31.3) | 17/82 (20.7) |
| Post-secondary, non-tertiary education | 231/1595 (14.5) | 159/864 (18.4) | 0 (0) | 19/112 (17) | 45/284 (15.8) | 8/82 (9.8) |
| Tertiary education | 440/1595 (27.6) | 347/864 (40.2) | 0 (0) | 20/112 (17.9) | 62/284 (21.8) | 11/82 (13.4) |
| **Health insurance** | | | | | |  |
| None | 69/1724 (4) | 0 (0) | 0 (0) | 0 (0) | 0 (0) | 69/93 (74.2) |
| Free state medical aid | 24/1724 (1.4) | 0 (0) | 0 (0) | 0 (0) | 0 (0) | 24/93 (25.8) |
| Basic health insurance | 342/1724 (19.9) | 136/943 (14.4) | 59/272 (21.7) | 41/116 (35.3) | 106/300 (35.3) | 0 (0) |
| Supplemental health insurance | 1289/1724 (74.8) | 807/943 (85.6) | 213/272 (78.3) | 75/116 (64.6) | 194/300 (64.7 | 0 (0) |
| **Income** | | | | | |  |
| None | 168/1700 (9.9) | 0 (0) | 0 (0) | 0 (0) | 117/296 (39.5) | 51/91 (56) |
| Minimum welfare | 184/1700 (10.8) | 0 (0) | 0 (0) | 0 (0) | 179/296 (60.5) | 5/91 (5.5) |
| Unemployment allocation | 84/1700 (4.9) | 56/934 (6) | 11/263 (4.2) | 17/114 (14.9) | 0 (0) | 0 (0) |
| Income from employment or pension | 1264/1700 (74.4) | 879/934 (94) | 253/263 (95.8) | 97/114 (85.1) | 0 (0) | 35/91 (38.5) |
| **Housing** | | | | | |  |
| Living on the street | 30/1726 (1.7) | 0 (0) | 0 (0) | 1/115 (0.9) | 14/300 (4.7) | 15/92 (16.3) |
| Shelter/hotel/hostel | 68/1726 (3.9) | 0 (0) | 0 (0) | 21/115 (18.2) | 35/300 (11.7) | 12/92 (13) |
| Housing by relatives | 219/1726 (12.7) | 0 (0) | 0 (0) | 93/115 (80.9) | 93/300 (31.1) | 33/92 (35.9) |
| Personal accommodation | 1409/1726 (81.6) | 949/949 (100) | 271/271 (100) | 0 (0) | 157/300 (52.5) | 32/92 (34.8) |

Abbreviations: IQR, interquartile range.

Table S2. Patients’ characteristics and outcomes, by inclusion center

| **Variable** | **Patients, No/total No (%)** | | | | | | | |
| --- | --- | --- | --- | --- | --- | --- | --- | --- |
|  | **Saint-Denis**  **Gen. hospital**  **n=212** | **Corbeil-Essonnes**  **Gen. hospital**  **n=327** | **Creteil**  **U. hospital**  **n=139** | **Paris 10^th^**  **U. hospital**  **n=254** | **Colombes**  **U. hospital**  **n=77** | **Longjumeau**  **Gen. hospital**  **n=149** | **Jossigny**  **Gen. hospital**  **n=304** | **Boulogne**  **U. hospital**  **n=286** |
| Hospital area poverty rate, % | 35 | 26 | 17 | 17 | 16 | 15 | 12 | 9 |
| **Demographics** | | | | | | | | |
| Age, median [IQR], y | 59.2 [43.2–70.6] | 63.1 [46.6–74.4] | 58.5 [44.2–71.2] | 56.8 [42–71] | 60.3 [38.6–72.6] | 62 [45.2–75.6] | 66.3 [55.2–76.8] | 67.9 [53.9–76.8] |
| Female sex | 93/212 (43.9) | 126/327 (38.5) | 46/139 (33.1) | 85/254 (33.5) | 34/77 (44.2) | 53/149 (35.6) | 117/304 (38.5) | 100/286 (35) |
| French nationality | 109/212 (51.4) | 259/327 (79.2) | 109/139 (78.4) | 159/254 (62.6) | 62/77 (80.5) | 118/149 (79.2) | 264/304 (86.8) | 246/286 (86) |
| **Socioeconomic characteristics** | | | | | | | | |
| Housing deprivation ^a^ | 56/209 (26.8) | 58/309 (18.8) | 29/139 (20.9) | 60/254 (23.6) | 25/77 (32.5) | 30/149 (20.1) | 34/304 (11.2) | 25/286 (8.7) |
| Income deprivation ^b^ | 63/197 (32) | 64/305 (21) | 29/139 (20.9) | 67/254 (26.4) | 15/77 (19.5) | 32/149 (21.5) | 51/304 (16.8) | 31/274 (11.3) |
| Health insurance deprivation ^c^ | 27/209 (12.9) | 11/323 (3.4) | 5/126 (4) | 26/252 (10.3) | 2/77 (2.6) | 4/149 (2.7) | 3/301 (1) | 15/283 (5.3) |
| Education deprivation ^d^ | 100/187 (53.5) | 86/287 (30) | 29/128 (22.7) | 40/252 (15.9) | 21/76 (27.6) | 44/149 (29.5) | 90/304 (29.6) | 18/212 (8.5) |
| Cumulative deprivation |  |  |  |  |  |  |  |  |
| 0 | 70/212 (33) | 163/327 (49.8) | 82/139 (59) | 156/254 (61.4) | 34/77 (44.2) | 74/149 (49.7) | 166/304 (54.6) | 213/286 (74.5) |
| 1 | 66/212 (31.1) | 105/327 (32.1) | 30/139 (21.6) | 41/254 (16.1) | 28/77 (36.4) | 50/149 (33.6) | 107/304 (35.2) | 49/286 (17.1) |
| 2 | 48/212 (22.6) | 40/327 (12.2) | 18/139 (12.9) | 32/254 (12.6) | 10/77 (13) | 16/149 (10.7) | 22/304 (7.2) | 15/286 (5.2) |
| ≥ 3 | 28/212 (13.2) | 19/327 (5.8) | 9/139 (6.5) | 25/254 (10.2) | 5/77 (6.5) | 9/149 (6) | 9/304 (3) | 9/286 (3.1) |
| Socioeconomic phenotypes |  |  |  |  |  |  |  |  |
| Phenotype A | 70/212 (33) | 163/327 (49.8) | 82/139 (59) | 156/254 (61.4) | 34/77 (44.2) | 74/149 (49.7) | 166/304 (54.6) | 213/286 (74.5) |
| Phenotype B | 50/212 (23.6) | 65/327 (19.9) | 15/139 (10.8) | 12/254 (4.7) | 13/77 (16.9) | 27/149 (18.1) | 72/304 (23.7) | 19/286 (6.6) |
| Phenotype C | 18/212 (8.5) | 25/327 (7.6) | 12/139 (8.6) | 12/254 (4.7) | 13/77 (16.9) | 16/149 (10.7) | 13/304 (4.3) | 8/286 (2.8) |
| Phenotype D | 47/212 (22.2) | 63/327 (19.3) | 25/139 (18) | 48/254 (18.9) | 15/77 (19.5) | 28/149 (18.8) | 50/304 (16.4) | 31/286 (10.8) |
| Phenotype E | 27/212 (12.7) | 11/327 (3.4) | 5/139 (3.6) | 26/254 (10.2) | 2/77 (2.6) | 4/149 (2.7) | 3/304 (1) | 15/286 (5.2) |

**Table S2. Continued**

| **Variable** | **Patients, No/total No (%)** | | | | | | | |
| --- | --- | --- | --- | --- | --- | --- | --- | --- |
|  | **Saint-Denis**  **Gen. hospital**  **n=212** | **Corbeil-Essonnes**  **Gen. hospital**  **n=327** | **Creteil**  **U. hospital**  **n=139** | **Paris 10^th^**  **U. hospital**  **n=254** | **Colombes**  **U. hospital**  **n=77** | **Longjumeau**  **Gen. hospital**  **n=149** | **Jossigny**  **Gen. hospital**  **n=304** | **Boulogne**  **U. hospital**  **n=286** |
| **Preadmission status** | | | | | | | | |
| Clinical Frailty Scale score ≥ 5 | 40/212 (18.9) | 71/327 (21.7) | 21/139 (15.1) | 36/254 (14.2) | 19/77 (24.7) | 24/149 (16.1) | 91/304 (29.9) | 86/286 (30.1) |
| Charlson comorbidity index ≥ 1 | 109/209 (52.2) | 183/324 (56.5) | 85/139 (61.2) | 121/251 (48.2) | 44/71 (62) | 69/149 (46.3) | 193/304 (63.5) | 172/286 (60.1) |
| **ICU admission** | | | | | | | | |
| SAPS 2 without age, median [IQR], points | 28 [19–47] | 17 [6–29] | 19 [12–34] | 28 [18–41] | 21 [15–37] | 24 [15–38] | 28 [17.5–48] | 23 [12–36] |
| SOFA score, median [IQR], points | 3 [1–6] | 3 [1–5] | 4 [2–5] | 4 [2–7] | 3 [2–5] | 3 [1–6] | 4 [2–8] | 3 [1–6] |
| **Supportive care** | | | | | | | | |
| Use of invasive ventilation | 62/212 (29.4) | 93/327 (28.4) | 44/139 (31.7) | 79/254 (31.1) | 21/77 (27.3) | 46/149 (30.9) | 98/304 (32.2) | 87/286 (30.4) |
| Use of vasopressors | 33/209 (15.8) | 62/327 (19) | 26/139 (18.7) | 72/254 (28.3) | 17/77 (22.1) | 30/149 (20.1) | 105/304 (34.5) | 79/286 (27.6) |
| Need for RRT | 20/212 (9.5) | 17/327 (5.2) | 15/139 (10.8) | 29/254 (11.4) | 5/77 (6.5) | 5/149 (3.4) | 36/304 (11.8) | 22/286 (7.7) |
| **Outcomes** | | | | | | | | |
| ICU length of stay, median [IQR], d | 4 [3–8] | 5 [3–7] | 5 [4–10] | 4 [2–7] | 5 [3–7] | 4 [3–7] | 4 [3–7] | 4 [3–7] |
| Hospital length of stay, median [IQR], d | 9 [4–21.5] | 10 [6–16] | 13 [7–24] | 7 [2–14] | 8 [4–15] | 8 [3–18] | 9 [5–18] | 10 [4–19] |
| ICU mortality | 27/212 (12.7) | 18/327 (5.5) | 17/139 (12.2) | 39/254 (15.4) | 10/77 (13) | 14/149 (9.4) | 52/304 (17.1) | 45/286 (15.7) |
| Day-180 mortality | 48/212 (22.6) | 53/327 (16.2) | 29/139 (20.9) | 63/254 (24.8) | 20/77 (26) | 25/149 (16.8) | 93/304 (30.6) | 73/286 (25.5) |

Abbreviations: d, days; ECLS, extra corporeal life support; Gen, general; ICU, intensive care unit; RRT, renal replacement therapy; SAPS, simplified acute physiology score; SOFA, sequential organ failure assessment; U, university; WLST, withdrawal or withholding of life sustaining therapies; y, years.

^a^ Defined as living on the street or shelter/hotel/hostel or housing by relatives

^b^ Defined as no income or minimum welfare

^c^ Defined as none or free state medical aid

^d^ Defined as primary education or below

Table S3. Evaluation of the association of each individual socioeconomic deprivation factor with 180-day mortality ^a^

|  | **Total effect estimation model ^a^** | | **Direct effect estimation model ^b^** | |
| --- | --- | --- | --- | --- |
| **Variable** | **HR [95% CI]** | ***P* value** | **HR [95% CI]** | ***P* value** |
| Housing deprivation ^c^ | 0.70 [0.51−0.98] | .03 | 0.76 [0.55−1.06] | .11 |
| Income deprivation ^d^ | 1.25 [0.92−1.69] | .15 | 1.08 [0.80−1.47] | .60 |
| Health insurance deprivation ^e^ | 1.24 [0.77−2.02] | .38 | 1.27 [0.78−2.07] | .33 |
| Education deprivation ^f^ | 0.89 [0.70−1.12] | .31 | 0.89 [0.71−1.13] | .34 |

Abbreviations: CI, Confidence Interval; HR, Hazard Ratio.

^a^ The association between each socioeconomic deprivation factor as a binary variable and 180-day mortality was individually tested with a multivariable Cox proportional hazard model stratified on center, and adjusted with the variables age, sex, and alcohol or opiate use.

^b^ The association between each socioeconomic deprivation factor as a binary variable and 180-day mortality was individually tested with a multivariable Cox proportional hazard model stratified on center, and adjusted with the variables age, sex, alcohol or opiate use, Charlson comorbidity index and admission SOFA score.

^c^ Defined as living on the street or shelter/hotel/hostel or housing by relatives

^d^ Defined as no income or minimum welfare

^e^ Defined as no health insurance or free state medical aid

^f^ Defined as primary education or below

Table S4. Evaluation of the association of cumulative socioeconomic deprivation with 180-day mortality

|  | **Total effect estimation model** | | **Direct effect estimation model** | |
| --- | --- | --- | --- | --- |
| **Variable** | **HR [95% CI]** | ***P* value** | **HR [95% CI]** | ***P* value** |
| Cumulative socioeconomic deprivations |  | .71 |  | .74 |
| 0 | Reference |  | Reference |  |
| 1 | 0.89 [0.71–1.13] |  | 0.98 [0.77–1.24] |  |
| 2 | 0.86 [0.59–1.26] |  | 0.87 [0.60–1.27] |  |
| 3 | 0.83 [0.45–1.53] |  | 0.73 [0.39–1.34] |  |
| 4 | 1.36 [0.59–3.10] |  | 1.29 [0.56–2.99] |  |
| Age, per 1 year increment | 1.04 [1.03–1.05] | <.001 | 1.03 [1.03–1.04] | <.001 |
| Female sex | 0.63 [0.51–0.79] | <.001 | 0.83 [0.66–1.04] | .10 |
| Alcohol or opiate use | 0.92 [0.71–1.20] | .55 | 0.91 [0.70–1.19] | .49 |
| Charlson comorbidity index ≥ 1 | - | - | 1.34 [1.05–1.71] | .02 |
| Admission SOFA score, per point | - | - | 1.23 [1.20–1.25] | <.001 |

Abbreviations: CI, confidence interval; HR, hazard ratio; SOFA, sequential organ failure assessment.

A cox proportional hazard model stratified on inclusion center was applied.

Table S5. Multivariable analyses of factors associated with 180-day mortality in patients with suboptimal clustering characteristics reclassified to the nearest neighboring cluster

|  | **Total effect estimation model** | | **Direct effect estimation model** | |
| --- | --- | --- | --- | --- |
| **Variable** | **HR [95% CI]** | ***P* value** | **HR [95% CI]** | ***P* value** |
| Socioeconomic phenotype |  | .11 |  | .57 |
| Phenotype A | Reference |  | Reference |  |
| Phenotype B | 0.85 [0.65–1.12] |  | 0.94 [0.72–1.24] |  |
| Phenotype C | 0.53 [0.31–0.90] |  | 0.66 [0.39–1.12] |  |
| Phenotype D | 1.09 [0.78–1.51] |  | 0.97 [0.70–1.35] |  |
| Phenotype E | 1.13 [0.68–1.86] |  | 1.16 [0.70–1.92] |  |
| Age, per 1 year increment | 1.04 [1.04–1.05] | <.001 | 1.03 [1.03–1.04] | <.001 |
| Female sex | 0.63 [0.50–0.78] | <.001 | 0.83 [0.66–1.04] | .11 |
| Alcohol or opiate use | 0.91 [0.70–1.18] | .47 | 0.90 [0.69–1.17] | .42 |
| Charlson comorbidity index ≥ 1 | - | - | 1.34 [1.05–1.70] | .02 |
| Admission SOFA score, per point | - | - | 1.22 [1.20–1.25] | <.001 |

Abbreviations: CI, confidence interval; HR, hazard ratio; SOFA, sequential organ failure assessment.

A cox proportional hazard model stratified on inclusion center was applied.

Table S6. Multivariable analyses of factors associated with 180-day mortality, without adjustment on alcohol and opiate use

|  | **Total effect estimation model** | | **Direct effect estimation model** | |
| --- | --- | --- | --- | --- |
| **Variable** | **HR [95% CI]** | ***P* value** | **HR [95% CI]** | ***P* value** |
| Socioeconomic phenotype |  | .14 |  | .59 |
| Phenotype A | Reference |  | Reference |  |
| Phenotype B | 0.86 [0.65–1.13] |  | 0.93 [0.71–1.23] |  |
| Phenotype C | 0.56 [0.34–0.94] |  | 0.70 [0.42–1.17] |  |
| Phenotype D | 1.08 [0.78–1.50] |  | 0.97 [0.70–1.34] |  |
| Phenotype E | 1.19 [0.73–1.95] |  | 1.22 [0.74–2.00] |  |
| Age, per 1 year increment | 1.04 [1.04–1.05] | <.001 | 1.03 [1.03–1.04] | <.001 |
| Female sex | 0.64 [0.51–0.79] | <.001 | 0.84 [0.67–1.05] | .13 |
| Charlson comorbidity index ≥ 1 | - | - | 1.33 [1.05–1.70] | .02 |
| Admission SOFA score, per point | - | - | 1.22 [1.20–1.25] | <.001 |

Abbreviations: CI, Confidence Interval; HR, Hazard Ratio; SOFA, Sequential Organ Failure Assessment.

A cox proportional hazard model stratified on inclusion center was applied.

Table S7. Diagnosis of communicable, maternal, neonatal and nutritional diseases at ICU discharge for the complete population and according to socioeconomic phenotypes ^a^

| **Variable** | **Patients, No (%)** | | | | | | ***P* value** |
| --- | --- | --- | --- | --- | --- | --- | --- |
|  | **All patients**  **n=1656 ^b^** | **Socioeconomic phenotypes** | | | | |  |
|  |  | **A**  **n=905 ^b^** | **B**  **n=255 ^b^** | **C**  **n=111 ^b^** | **D**  **n=294 ^b^** | **E**  **n=91 ^b^** |  |
| **Respiratory infections and tuberculosis** | **205 (12.4)** | **108 (11.9)** | **37 (14.5)** | **10 (9)** | **36 (12.2)** | **14 (15.4)** | **.53** |
| Lower respiratory infections | 184 (11.1) | 100 (11) | 32 (12.5) | 8 (7.2) | 31 (10.5) | 13 (14.3) | - |
| Upper respiratory infections | 15 (0.9) | 8 (0.9) | 3 (1.2) | 1 (0.9) | 3 (1) | 0 (0) |  |
| Tuberculosis | 6 (0.4) | 0 (0) | 2 (0.8) | 1 (0.9) | 2 (0.7) | 1 (1.1) |  |
| **Maternal and neonatal disorders** | **36 (2.2)** | **20 (2.2)** | **2 (0.8)** | **2 (1.8)** | **7 (2.4)** | **5 (5.5)** | **.13** |
| Maternal disorders | 36 (2.2) | 20 (2.2) | 2 (0.8) | 2 (1.8) | 7 (2.4) | 5 (5.5) | **-** |
| **Other infectious diseases** | **32 (1.9)** | **20 (2.2)** | **5 (2)** | **2 (1.8)** | **3 (1)** | **2 (2.2)** | **.79** |
| Other un specified infectious diseases | 22 (1.3) | 12 (1.3) | 3 (1.2) | 2 (1.8) | 3 (1) | 2 (2.2) | - |
| Meningitis | 6 (0.4) | 4 (0.4) | 2 (0.8) | 0 (0) | 0 (0) | 0 (0) |  |
| Encephalitis | 3 (0.2) | 3 (0.3) | 0 (0) | 0 (0) | 0 (0) | 0 (0) |  |
| Acute hepatitis | 1 (0.1) | 1 (0.1) | 0 (0) | 0 (0) | 0 (0) | 0 (0) |  |
| **Neglected tropical diseases and malaria** | **3 (0.2)** | **2 (0.2)** | **0 (0)** | **0 (0)** | **0 (0)** | **1 (1.1)** | **-** |
| Malaria | 3 (0.2) | 2 (0.2) | 0 (0) | 0 (0) | 0 (0) | 1 (1.1) | **-** |
| **Enteric infections** | **2 (0.1)** | **1 (0.1)** | **1 (0.4)** | **0 (0)** | **0 (0)** | **0 (0)** | **-** |
| Diarrheal diseases | 2 (0.1) | 1 (0.1) | 1 (0.4) | 0 (0) | 0 (0) | 0 (0) | **-** |

^a^ Classification adapted from the Global Burden of Diseases study (<https://www.thelancet.com/gbd>)

^b^ Patients with available diagnosis at ICU discharge

Table S8. Diagnosis of Injuries at ICU discharge for the complete population and according to socioeconomic phenotypes ^a^

| **Variable** | **Patients, No (%)** | | | | | | ***P* value** |
| --- | --- | --- | --- | --- | --- | --- | --- |
|  | **All patients**  **n=1656 ^b^** | **Socioeconomic phenotypes** | | | | |  |
|  |  | **A**  **n=905 ^b^** | **B**  **n=255 ^b^** | **C**  **n=111 ^b^** | **D**  **n=294 ^b^** | **E**  **n=91 ^b^** |  |
| **Self-harm and interpersonal violence** | **207 (12.5)** | **107 (11.8)** | **17 (6.7)** | **16 (14.4)** | **48 (16.3)** | **19 (20.9)** | **<.01** |
| Self-harm | 207 (12.5) | 107 (11.8) | 17 (6.7) | 16 (14.4) | 48 (16.3) | 19 (20.9) | - |
| **Unintentional injuries** | **163 (9.8)** | **90 (9.9)** | **30 (11.8)** | **11 (9.9)** | **24 (8.2)** | **8 (8.8)** | **.71** |
| Other unintentional injuries | 111 (6.7) | 61 (6.7) | 19 (7.5) | 7 (6.3) | 18 (6.1) | 6 (6.6) | - |
| Foreign body | 40 (2.4) | 19 (2.1) | 10 (3.9) | 4 (3.6) | 5 (1.7) | 2 (2.2) |  |
| Adverse effects of medical treatment | 11 (0.7) | 9 (1) | 1 (0.4) | 0 (0) | 1 (0.3) | 0 (0) |  |
| Fire, heat, and hot substances | 1 (0.1) | 1 (0.1) | 0 (0) | 0 (0) | 0 (0) | 0 (0) |  |

^a^ Classification adapted from the Global Burden of Diseases study (<https://www.thelancet.com/gbd>)

^b^ Patients with available diagnosis at ICU discharge

Table S9. Diagnosis of non-communicable diseases at ICU discharge for the complete population and according to socioeconomic phenotypes ^a^

| **Variable** | **Patients, No (%)** | | | | | | ***P* value** |
| --- | --- | --- | --- | --- | --- | --- | --- |
|  | **All patients**  **n=1656 ^b^** | **Socioeconomic phenotypes** | | | | |  |
|  |  | **A**  **n=905 ^b^** | **B**  **n=255 ^b^** | **C**  **n=111 ^b^** | **D**  **n=294 ^b^** | **E**  **n=91 ^b^** |  |
| **Cardiovascular diseases** | **295 (17.8)** | **175 (19.3)** | **63 (24.7)** | **9 (8.1)** | **34 (11.6)** | **14 (15.4)** | **<.01** |
| Other cardiovascular and circulatory diseases | 178 (10.7) | 102 (11.3) | 41 (16.1) | 7 (6.3) | 19 (6.5) | 9 (9.9) | - |
| Ischemic heart disease | 62 (3.7) | 41 (4.5) | 9 (3.5) | 2 (1.8) | 8 (2.7) | 2 (2.2) |  |
| Stroke | 42 (2.5) | 25 (2.8) | 9 (3.5) | 0 (0) | 5 (1.7) | 3 (3.3) |  |
| Endocarditis | 9 (0.5) | 5 (0.6) | 3 (1.2) | 0 (0) | 1 (0.3) | 0 (0) |  |
| Cardiomyopathy and myocarditis | 3 (0.2) | 1 (0.1) | 1 (0.4) | 0 (0) | 1 (0.3) | 0 (0) |  |
| Hypertensive heart disease | 1 (0.1) | 1 (0.1) | 0 (0) | 0 (0) | 0 (0) | 0 (0) |  |
| **Other non-communicable diseases** | **176 (10.6)** | **93 (10.3)** | **23 (9)** | **13 (11.7)** | **41 (13.9)** | **6 (6.6)** | **.21** |
| Endocrine, metabolic, blood, and immune disorders | 88 (5.3) | 56 (6.2) | 10 (3.9) | 5 (4.5) | 15 (5.1) | 2 (2.2) | - |
| Urinary diseases and male infertility | 54 (3.3) | 27 (3) | 11 (4.3) | 2 (1.8) | 10 (3.4) | 4 (4.4) |  |
| Haemoglobinopathies and hemolytic anemias | 34 (2.1) | 10 (1.1) | 2 (0.8) | 6 (5.4) | 16 (5.4) | 0 (0) |  |
| **Digestive diseases** | **162 (9.8)** | **97 (10.7)** | **33 (12.9)** | **7 (6.3)** | **20 (6.8)** | **5 (5.5)** | **.04** |
| Upper digestive system diseases | 78 (4.7) | 47 (5.2) | 18 (7.1) | 4 (3.6) | 6 (2) | 3 (3.3) | - |
| Other digestive diseases | 35 (2.1) | 25 (2.8) | 5 (2) | 0 (0) | 5 (1.7) | 0 (0) |  |
| Pancreatitis | 15 (0.9) | 7 (0.8) | 1 (0.4) | 2 (1.8) | 3 (1) | 2 (2.2) |  |
| Gallbladder and biliary diseases | 14 (0.8) | 10 (1.1) | 2 (0.8) | 1 (0.9) | 1 (0.3) | 0 (0) |  |
| Vascular intestinal disorders | 7 (0.4) | 3 (0.3) | 3 (1.2) | 0 (0) | 1 (0.3) | 0 (0) |  |
| Cirrhosis and other chronic liver diseases | 6 (0.4) | 2 (0.2) | 2 (0.8) | 0 (0) | 2 (0.7) | 0 (0) |  |
| Paralytic ileus and intestinal obstruction | 5 (0.3) | 2 (0.2) | 1 (0.4) | 0 (0) | 2 (0.7) | 0 (0) |  |
| Inguinal, femoral, and abdominal hernia | 2 (0.1) | 1 (0.1) | 1 (0.4) | 0 (0) | 0 (0) | 0 (0) |  |

**Table S9. Continued ^a^**

| **Variable** | **Patients, No (%)** | | | | | | ***P* value** |
| --- | --- | --- | --- | --- | --- | --- | --- |
|  | **All patients**  **n=1656 ^b^** | **Socioeconomic phenotypes** | | | | |  |
|  |  | **A**  **n=905 ^b^** | **B**  **n=255 ^b^** | **C**  **n=111 ^b^** | **D**  **n=294 ^b^** | **E**  **n=91 ^b^** |  |
| Paralytic ileus and intestinal obstruction | 5 (0.3) | 2 (0.2) | 1 (0.4) | 0 (0) | 2 (0.7) | 0 (0) | - |
| Inguinal, femoral, and abdominal hernia | 2 (0.1) | 1 (0.1) | 1 (0.4) | 0 (0) | 0 (0) | 0 (0) |  |
| **Chronic respiratory diseases** | **142 (8.6)** | **69 (7.6)** | **21 (8.2)** | **25 (22.5)** | **24 (8.2)** | **3 (3.3)** | **<.01** |
| Chronic obstructive pulmonary disease | 110 (6.6) | 53 (5.9) | 18 (7.1) | 20 (18) | 19 (6.5) | 0 (0) | - |
| Asthma | 24 (1.4) | 9 (1) | 3 (1.2) | 4 (3.6) | 5 (1.7) | 3 (3.3) |  |
| Interstitial lung disease and pulmonary sarcoidosis | 8 (0.5) | 7 (0.8) | 0 (0) | 1 (0.9) | 0 (0) | 0 (0) |  |
| **Diabetes and kidney diseases** | **96 (5.8)** | **49 (5.4)** | **9 (3.5)** | **9 (8.1)** | **23 (7.8)** | **6 (6.6)** | **.20** |
| Diabetes mellitus | 54 (3.3) | 21 (2.3) | 4 (1.6) | 7 (6.3) | 19 (6.5) | 3 (3.3) | - |
| Acute glomerulonephritis | 42 (2.5) | 28 (3.1) | 5 (2) | 2 (1.8) | 4 (1.4) | 3 (3.3) |  |
| **Neurological disorders** | **79 (4.8)** | **35 (3.9)** | **6 (2.4)** | **6 (5.4)** | **25 (8.5)** | **7 (7.7)** | **<.01** |
| Epilepsy | 71 (4.3) | 31 (3.4) | 6 (2.4) | 5 (4.5) | 23 (7.8) | 6 (6.6) | - |
| Multiple sclerosis | 5 (0.3) | 3 (0.3) | 0 (0) | 1 (0.9) | 1 (0.3) | 0 (0) |  |
| Other neurological disorders | 3 (0.2) | 1 (0.1) | 0 (0) | 0 (0) | 1 (0.3) | 1 (1.1) |  |
| **Skin and subcutaneous diseases** | **27 (1.6)** | **16 (1.8)** | **5 (2)** | **0 (0)** | **5 (1.7)** | **1 (1.1)** | **-** |
| Bacterial skin diseases | 26 (1.6) | 15 (1.7) | 5 (2) | 0 (0) | 5 (1.7) | 1 (1.1) | - |
| Other skin and subcutaneous diseases | 1 (0.1) | 1 (0.1) | 0 (0) | 0 (0) | 0 (0) | 0 (0) |  |
| **Neoplasms** | **22 (1.3)** | **16 (1.8)** | **3 (1.2)** | **0 (0)** | **3 (1)** | **0 (0)** | **-** |
| Neoplasms | 22 (1.3) | 16 (1.8) | 3 (1.2) | 0 (0) | 3 (1) | 0 (0) | - |
| **Musculoskeletal disorders** | **8 (0.5)** | **7 (0.8)** | **0 (0)** | **1 (0.9)** | **0 (0)** | **0 (0)** | **-** |
| Osteoarthritis | 8 (0.5) | 7 (0.8) | 0 (0) | 1 (0.9) | 0 (0) | 0 (0) | - |
| **Nutritional deficiencies** | **1 (0.1)** | **0 (0)** | **0 (0)** | **0 (0)** | **1 (0.3)** | **0 (0)** | **-** |
| Other nutritional deficiencies | 1 (0.1) | 0 (0) | 0 (0) | 0 (0) | 1 (0.3) | 0 (0) | - |

^a^ Classification adapted from the Global Burden of Diseases study (<https://www.thelancet.com/gbd>) ; ^b^ Patients with available diagnosis at ICU discharge
